# Recovery from pneumonia requires efferocytosis which is impaired in smokers and those with low body mass index and enhanced by statins

**DOI:** 10.1136/thoraxjnl-2016-208505

**Published:** 2016-07-28

**Authors:** Daniel G Wootton, Peter J Diggle, Joanne Court, Odiri Eneje, Lynne Keogan, Laura Macfarlane, Sarah Wilks, Mark Woodhead, Stephen B Gordon

**Affiliations:** 1 Institute of Infection and Global Health, University of Liverpool, Liverpool, UK; 2 Department of Respiratory Research, Aintree University Hospital NHS Foundation Trust, Liverpool, UK; 3 CHICAS, Lancaster University Medical School, Lancaster University, Lancaster, UK; 4 Department of Clinical Sciences, Liverpool School of Tropical Medicine, Liverpool, UK; 5 Department of Respiratory Medicine, Central Manchester University Hospitals NHS Foundation Trust, Manchester, UK; 6 Manchester Academic Health Science Centre and Faculty of Medical and Human Sciences, University of Manchester, Manchester, UK

**Keywords:** Macrophage Biology, Pneumonia, Neutrophil Biology, Tobacco and the lung

## Abstract

**Background:**

Efferocytosis (the phagocytosis of apoptotic self cells) is a key mechanism in the resolution of inflammatory processes such as community-acquired pneumonia (CAP). Efferocytosis therefore represents a modifiable target for therapy aimed at enhancing intrinsic recovery mechanisms. It is currently not known which patients recovering from CAP would mostly benefit from a strategy aimed at enhancing efferocytosis.

**Methods:**

We recruited a cohort of patients with CAP admitted to a hospital in Liverpool. One month into recovery, subjects were invited for research bronchoscopy and bronchoalveolar lavage. An ex vivo efferocytosis assay was performed by challenging alveolar macrophages with autologous, apoptotic neutrophils. The percentage of alveolar macrophages that had undergone efferocytosis was determined by flow cytometry. We conducted a multivariable regression using a linear mixed effects model to determine which clinical parameters were most closely associated with efferocytosis.

**Results:**

We observed high rates of comorbidity among this CAP cohort. Efferocytosis was measured in 22 subjects. We assessed multiple combinations of clinical parameters for association with efferocytosis and found the best-fitting model included an interaction between smoking status and prior statin use—smoking being associated with decreased efferocytosis and statin use with increased efferocytosis. These effects were modified by an association between efferocytosis and body mass index (BMI), such that as BMI increased so did efferocytosis.

**Conclusions:**

This is the first study to measure efferocytosis in patients recovering from CAP. The results suggest that smokers with low BMI have impaired efferocytosis and may benefit from a statin to boost recovery.

## Introduction

Among the 80% of patients who survive an admission with community-acquired pneumonia (CAP), a proportion suffers prolonged symptoms.^1^ During recovery from pneumonia, dead and dying neutrophils must be cleared. This phagocytosis of apoptotic ‘self’ cells, called efferocytosis, is defective in idiopathic pulmonary fibrosis and COPD.^2^ The efferocytosis of apoptotic neutrophils has been studied in pneumonic mice but not in humans recovering from CAP.^3^ In patients with CAP, clinical severity markers are associated with short-term mortality risk, but the association of these factors with efferocytosis following CAP is not known. If pre-CAP clinical factors were associated with efferocytosis, then pro-resolution therapy to reduce post-CAP adverse events would become a possibility. We conducted a prospective cohort study of adults hospitalised for CAP. We hypothesised that ex vivo efferocytosis of autologous apoptotic neutrophils by alveolar macrophages would vary in association with patient characteristics.

## Methods

Subjects recruited from two UK Hospitals between February 2011 and March 2013 had CAP (British Thoracic Society definition), were aged >16 years and were recruited within 24 hours of their first dose of in-hospital antibiotic. We excluded patients requiring invasive ventilation, requiring renal replacement therapy, with cystic fibrosis (CF), non-CF bronchiectasis, lung cancer, lung metastases, advanced cancer of any type, immunocompromise (including systemic corticosteroids), those treated palliatively or admitted within 14 days. At 1 month, subjects were invited for bronchoscopy with bronchoalveolar lavage (BAL) and were excluded if they were at increased risk of complications. BAL was performed as previously published with 200 ml saline instilled into the right middle lobe (RML) bronchus.^4^ BAL was filtered, pelleted, washed and resuspended in Iscove's Modified Dubecco's Medium (IMDM) with human AB serum and antibiotics. Cells were seeded into 48 well plates and incubated for 4 hours before the medium was replaced with antibiotic-free IMDM.

Ex vivo autologous apoptotic neutrophils were derived by published protocol.^5^ Prior to incubation, the neutrophils were divided into two aliquots: one stained green and the other unstained. The efferocytosis assay was a modification of published protocols.^6^ After overnight incubation, the media was removed from the macrophages and unstained neutrophils, stained neutrophils or medium added to each well (see online supplementary figure S1). After 90 min co-culture, the medium was removed and the macrophages washed to remove uningested neutrophils. Macrophages were detached with cold phosphate-buffered saline (PBS), washed, quenched and then acquired on the cytometer.

10.1136/thoraxjnl-2016-208505.supp1 supplementary data

The flow cytometric gating strategy (see online supplementary figure S2) involved the separation of uningested neutrophils from macrophages by light scatter as previously published.^6^ Using R, associations with efferocytosis were analysed by multiple regression. The mean of efferocytosis experimental replicates was used for univariate analysis; then, a maximal linear mixed effects model was constructed using efferocytosis as the response variable, correlated (p>0.05) variables from the univariate analysis as fixed effects and ‘subject id’ as a random effect. The random effect enabled us to separate the stochastic variation associated with experimental technique from between subject variations. Backwards elimination derived the minimum set of explanatory variables required to give a statistically acceptable fit.

## Results

**Figure 1:**
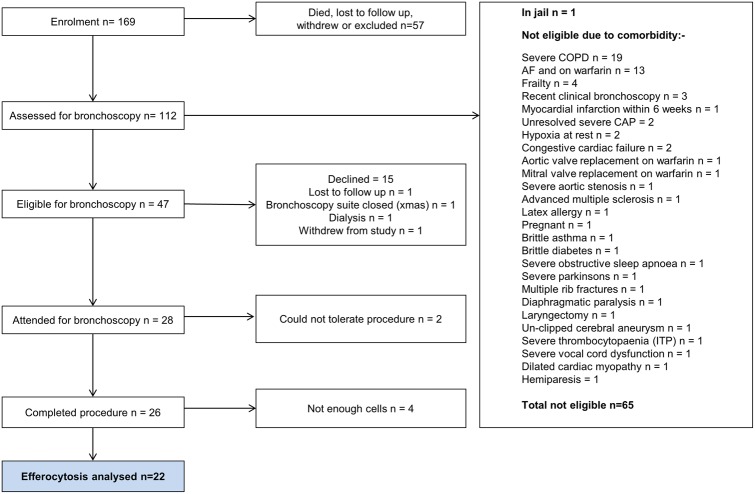
Bronchoscopy flow chart. Approximately half of the subjects assessed were deemed ineligible for research bronchoscopy—the majority of those being excluded on safety grounds due to comorbidity. CAP, community-acquired pneumonia

**Figure 2:**
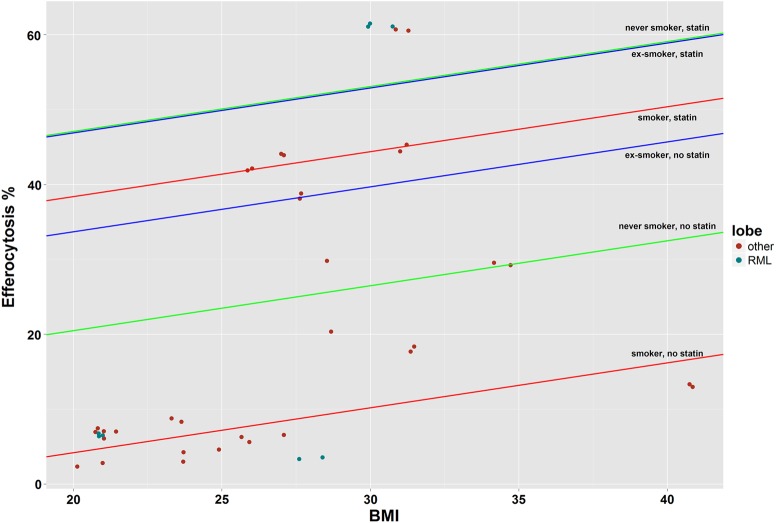
Interactions between covariates in the linear mixed effects model. The result of the linear regression was a model that included the interaction of smoking and statin with adjustment for body mass index (BMI). The efferocytosis values fitted by the model (including all replicates) are on the Y-axis, with BMI on the X-axis. Points are ‘jittered’ to avoid overlap. The modelled interactions of each level of smoking and statin are represented by six straight lines. These lines are displayed in colour pairs based on smoking status. The y-intercepts of each line are derived from the model, as is the BMI-dependent slope of 0.6. From these lines, it can be seen that without statins the differences between smoking status are large, but with statins the differences are smaller. The magnitude of the statin effect appears greatest in smokers. There was no association between efferocytosis and sampling from the right middle lobe (RML).

Of 169 subjects recruited, efferocytosis was analysed in 22 (figure 1 and online supplementary tables S1 and S2). Univariate analysis (see online supplementary table S3) revealed a trend towards higher efferocytosis values with improved symptomatic recovery (see online supplementary figure S3), but no relationship with age. Smoking status, prior statin use, body mass index (BMI) and gender correlated with efferocytosis. Those variables were combined in a linear mixed effects model which left three variables with statistically significant effects: smoking status, prior statin use and BMI. As BMI increased, so did efferocytosis. Smoking was associated with lower rates of efferocytosis. Subjects who were taking statins had higher rates of efferocytosis. Analysis for interactions showed the model with the best data-fit included an interaction between smoking status and prior statin use, with adjustment for BMI (figure 2). The interaction was such that the statin-associated increase in efferocytosis was largest in those who were active smokers. The final model explained 42.6% of variation in the data, of which 90.1% was the difference between patients and 9.9% was within experimental replicates.

## Discussion

This is the first study of efferocytosis during recovery from CAP. After adjustment for BMI, the strongest associations with efferocytosis were smoking status and statin use prior to CAP.

Strengths of this study include the use of autologous neutrophils as a pathophysiologically appropriate apoptotic target for the efferocytosis assay, the use of linear mixed effects modelling to quantify the contributions of experimental and between-patient variations and the flow cytometric method used. Limitations include the small study size, lower median age and lower range of severity than that in clinical practice. It is not known if CAP in humans has an effect on efferocytosis or whether any such effect would be local or generalised, but we consistently lavaged the RML, and three of the patients were recovering from RML CAP. It is possible that recent local inflammation by the RML CAP may have affected rates of local efferocytosis; however, in our univariate analysis, there was no statistically significant difference in efferocytosis associated with RML involvement.

Previous studies have found that cigarette smoke affects molecular pathways that lead to the activation and membrane localisation of the enzyme Rac which facilitates the cytoskeletal rearrangements needed for efferocytosis.^7^ Statins cause the enzymes Rac and RhoA to sequester in the cytosol, resulting in increased efferocytosis of apoptotic neutrophils.^8^ These studies suggest smoking and statins have antagonistic effects on Rac1 and RhoA, and as a consequence opposite effects on efferocytosis, providing a possible mechanistic explanation for our finding of a negative association between efferocytosis and smoking, a positive association with statins and a statistical interaction between smoking and statin use.

We also showed a novel association between BMI and efferocytosis. Previous studies have shown that reduced CAP mortality is associated with high BMI^9^ and that low BMI is associated with increased risk of developing CAP.^10^ Differential rates of efferocytosis may explain these correlations.

Our study suggests that smokers with CAP and low BMI may benefit most from augmented efferocytosis; a statin would be an appropriate candidate for such a trial.
