# Supplementary material for: Recovery from pneumonia requires efferocytosis which is impaired in smokers and those with low body mass index and enhanced by statins
Source: Thorax. 2016 Jul 28;71(11):1052–4. doi: 10.1136/thoraxjnl-2016-208505 (PMC5099176; doi:10.1136/thoraxjnl-2016-208505)
Supplement: supplementary data [file thoraxjnl-2016-208505supp.pdf]

Supplementary table 1: Subject characteristics

Characteristic		Bronchoscopy Group n=22	No-Bronchoscopy Group n=147	P
Age, median (IQR)		51(42-58)	71(56-79)	**<0.0001**#
Male, n (%)		14(64)	74(50)	0.350*
Smoking, n (%)	Active	13(59)	50(36)	0.051*
	Quit	4(18)	62(45)	
	Never	5(22)	27(19)	
Modified Charlson comorbidity index	0	13(59)	43(29)	0.197*
	1	5(23)	64(44)	
	2	2(9)	16(11)	
	3	2(9)	15(10)	
	4	0	6(4)	
	5	0	2(1)	
	6	0	1(0.6)	
Prior statin use, n (%)		5(23)	52(36)	0.343*
COPD, n (%)		6(27)	67(46)	**0.009***
CURB65, n (%)	0	14(64)	26(18)	**<0.0001***
	1	2(9)	37(25)	
	2	5(23)	45(31)	
	3	1(4)	35(24)	
	4	0(0)	4(3)	
	5	0(0)	0(0)	
Presenting CRP, median (IQR)		188(135-316)	139(60-229)	**0.02**$
BMI, median (IQR)		27(24-30)	24(22-31)	0.8$
Influenza, n (%)		5(24)	13(15)	0.529*
Pneumococcal bacteraemia, n (%)		3(14)	5(4)	0.116*

Clinical characteristics were compared between study subjects who had and did not have efferocytosis measured. 'Influenza' data describe those whose influenza antibody titre climbed fourfold from presentation with CAP suggesting acute infection. Those who had a bronchoscopy and therefore had efferocytosis measured were younger and had a higher CRP at the time they presented with CAP.

Wilcoxon rank sum test

* Chi squared test

$ Welch's t test

Where data was incomplete for a particular variable, n is indicated below:-

Smoking status, no bronchoscopy group, n=139

Prior statin use, no-bronchoscopy group, n=146

CRP, no bronchoscopy group, n=144

BMI, no bronchoscopy group, n=109

Influenza, bronchoscopy group, n=21; no bronchoscopy group, n=86

Pneumococcal bacteraemia, no bronchoscopy group, n=137

Supplementary table 2: Efferocytosis raw data

Subject ID	Efferocytosis %
1	39.8, 41.5
2	64.6, 58.6
3	6.4
4	17.5, 21.0
5	26.9, 31.8
6	6.5, 5.3, 6.1, 3.0
7	41.8, 44.0
8	4.3, 8.6, 7.5
9	1.5
10	58.1, 59.8, 69.2
11	1.4, 3.0
12	7.6, 7.7, 5.8
13	3.0, 2.5
14	18.9
15	45.7, 44.0
16	9.7, 7.9
17	58.6, 58.3, 70.9, 61.8, 54.8, 53.2
18	31.7
19	36.2, 40.6
20	7.8, 4.7, 7.6, 7.7
21	11.6, 13.6
22	6.8, 8.2
	Median = 13.6 IQR = 37.6

Where a subject has multiple values in the efferocytosis column these indicate experimental replicates. The ability to perform replicates of the experiment was dependent on the number of cells obtained from the bronchoalveolar lavage.

Supplementary table 3: Univariate analysis of associations between clinical parameters and efferocytosis using linear modelling

Explanatory Variables	Regression coefficient	95% confidence interval	Adjusted R^2	*p*
Statin use	34.98	19.33 to 50.62	0.497	**0.0001**
Smoker	10.13	2.7 to 17.6	0.62	**0.01**
Ex-smoker	39.93	24.6 to 55.2		**<0.0001**
Never smoker	26.17	12.1 to 40.3		**0.0009**
Gender	19.7	2.3 to 37.12	0.18	**0.03**
BMI	1.77	0.116 to 3.43	0.167	**0.037**
Oral steroid use	-21.56	-47.26 to 4.14	0.09	0.096
Macrolide use	-21.03	-46.8 to 4.77	0.08	0.105
Admission CAP-sym score	-0.426	-1.1 to 0.25	0.03	0.203
COPD	-12.67	-33.10 to 7.76	0.03	0.21
Late apoptosis	-0.9	-2.49 to 0.69	0.019	0.25
Early apoptosis	-0.16	-0.55 to 0.23	-0.012	0.394
Combined apoptosis	-0.102	-0.44 to 0.24	-0.029	0.538
Pneumococcal blood culture positive	-0.42	-41 to 12.6	-0.03	0.57
Presenting pro-calcitonin	0.44	-1.17 to 2.05	-0.03	0.574
Inhaled steroid use	-4.85	-29.3 to 19.6	-0.041	0.683
Presenting CRP	-0.01	-0.08 to 0.06	-0.04	0.689

CURB65 1	3.62	-29.89-37.13		0.82
CURB65 2	8.83	-14.26-31.93	-0.09	0.43
CURB65 3	-14.58	-60.47-31.31		0.51
Age	-0.03	-0.64 to 0.58	-0.05	0.9
Influenza infection	-1.15	-24.2 to 21.9	-0.05	0.93
Middle lobe consolidation	0.81	-26.8 to 28.4	-0.05	0.95

For the univariate analysis the mean of each subjects efferocytosis experimental replicates was used.

Supplementary Figure 1. Ex-vivo efferocytosis

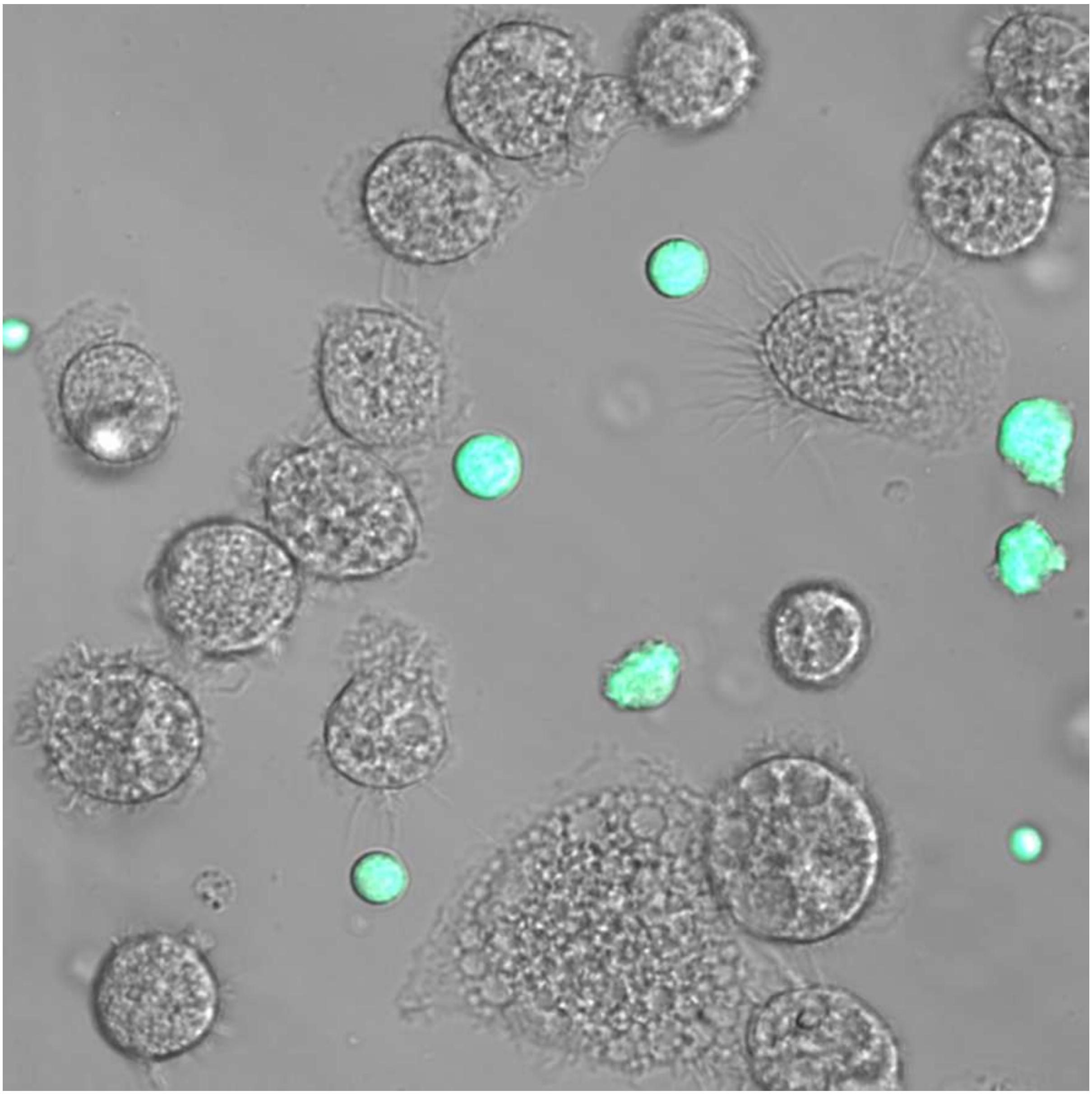

Alveolar macrophages obtained from subjects one month into their recovery from CAP are co-cultured for 90 minutes with autologous apoptotic neutrophils. For this image the ex-vivo assay was conducted in a dedicated cell culture chamber at 37°C, 5% CO_2 mounted on a confocal microscope and the image is a composite of simultaneously acquired bright-field and blue laser images. The neutrophils have been stained and fluoresce bright green. Macrophages can be seen extending their podocytes towards neutrophils prior to efferocytosis.

Supplementary Figure 2. Ex-vivo efferocytosis flow-cytometry gating strategy

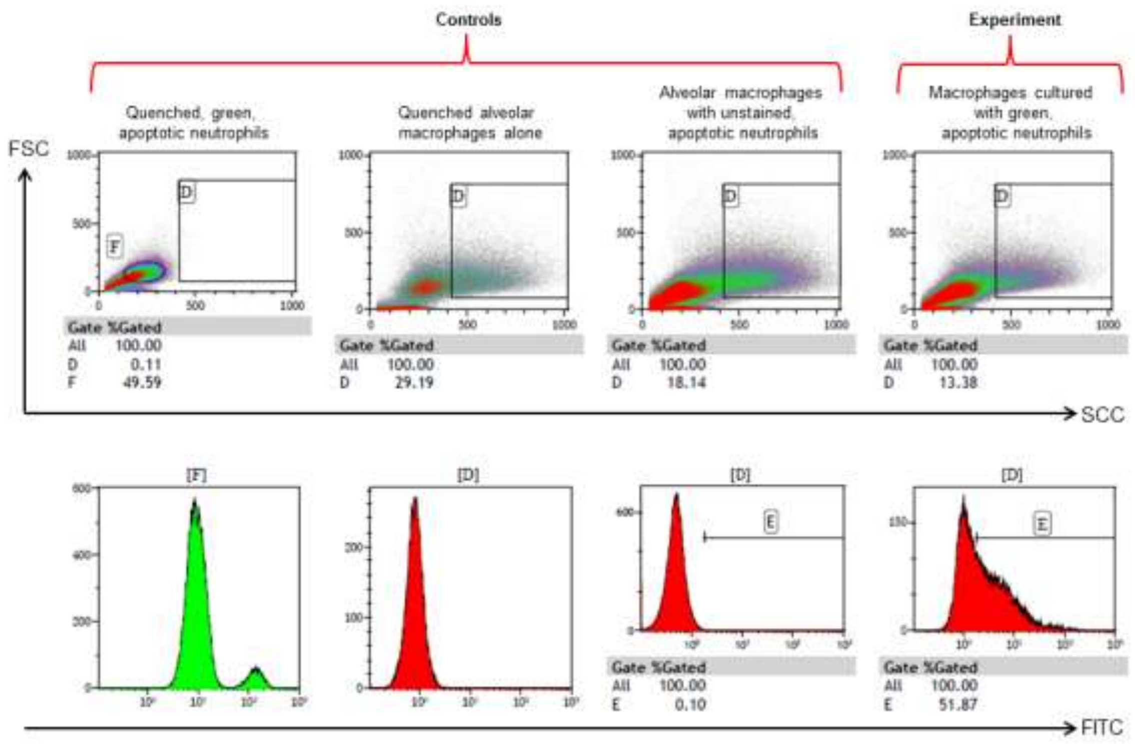

From left to right, the first 3 pairs of plots are controls run at the time of each patient's efferocytosis experiment.

Cells selected by light scatter in gate [F] are quenched, green stained neutrophils and their FITC fluorescence is seen in the histogram beneath.

Gate [D] excludes all but 0.11% of neutrophils but includes alveolar macrophages.

Gate [E] excludes all but 0.1% of macrophages which have been co-cultured with unstained apoptotic neutrophils as a control experiment.

In the pair of plots on the far right the macrophages are selected by light scatter in gate [D] and those macrophages that fall within gate [E] have a higher FITC signal than is seen in the control experiment. We infer that these $FITC^{high}$ macrophages have ingested green apoptotic neutrophils.

We express efferocytosis as [E] as a percentage of [D] – which in this experiment is 51.87%

Supplementary Figure 3. The relationship between symptom recovery and efferocytosis

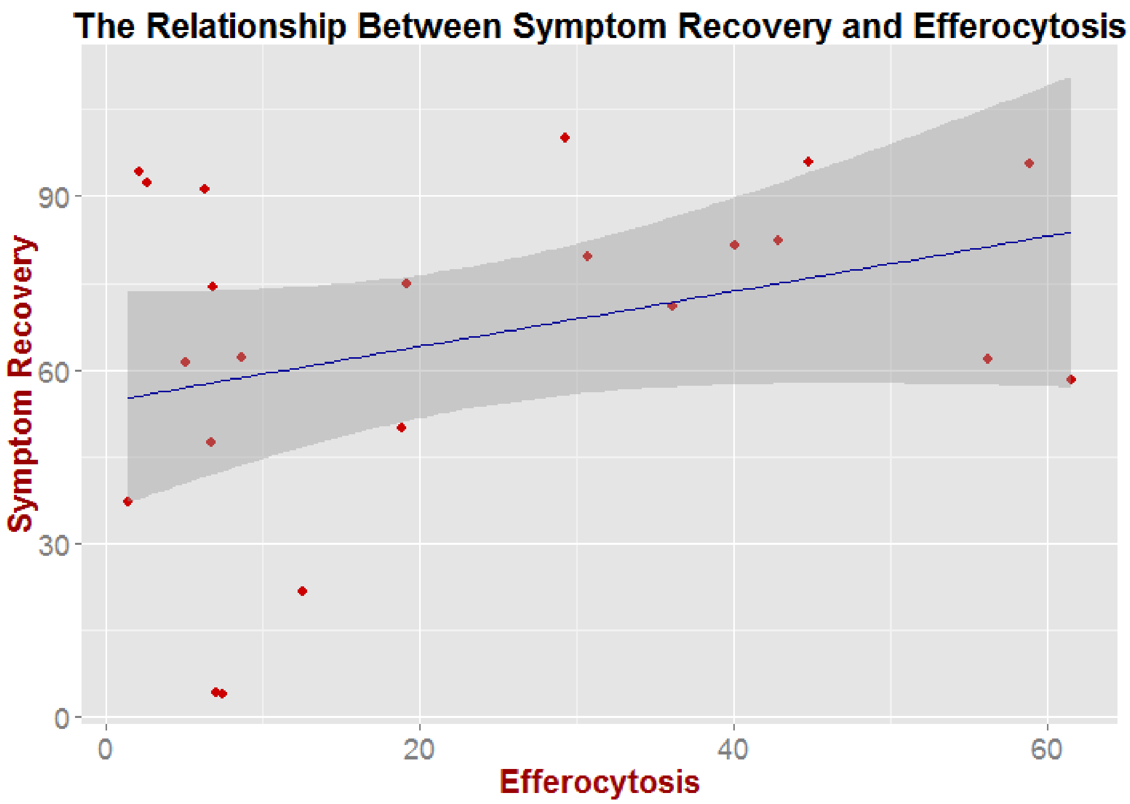

Symptoms were measured using the CAP-sym questionnaire. CAP-sym contains 18 questions and each question is phrased in a similar way, “in the last 24 hours how much have you been bothered by (e.g.) shortness of breath?” The subject can choose one of six possible answers and each answer carries a numerical score:

I do not have the symptom (scores 0)

not bothered at all (scores 1)

a little bothered (scores 2)

moderately bothered (scores 3)

bothered quite a bit (scores 4)

extremely bothered (scores 5)

If the scores for each of the 18 component answers are summed they come to a maximum score of 90 which represents the worst a patient could possibly feel with respect to these symptoms. The total score for the questionnaire therefore represents the burden of

pneumonia-related symptoms felt by that patient in the 24 hours preceding the completion of the questionnaire.

At the time of enrolment subjects conducted the CAP-sym questionnaire twice. The first iteration of the questionnaire represented their symptoms in the previous 24 hours i.e. the day of admission to hospital with CAP. The second was completed *thinking back 30 days* prior to admission and represented how they felt before the pneumonia began. CAP-sym was repeated at the one month follow-up visit just prior to bronchoscopy.

Recovery was calculated as the percentage improvement in CAP-sym score at one month compared to the CAP-sym score at presentation. This recovery measure is then plotted against the efferocytosis result for 22 subjects. Linear modelling revealed a non-statistically significant trend towards increasing recovery with increasing efferocytosis as displayed by the blue line (shaded area = 95% CI around the position of the line).
